# Characterisation of hepatic lipid signature distributed across the liver zonation using mass spectrometry imaging

**DOI:** 10.1016/j.jhepr.2023.100725

**Published:** 2023-03-09

**Authors:** Patcharamon Seubnooch, Matteo Montani, Sofia Tsouka, Emmanuelle Claude, Umara Rafiqi, Aurel Perren, Jean-Francois Dufour, Mojgan Masoodi

**Affiliations:** 1 Institute of Clinical Chemistry, Inselspital, Bern University Hospital, Bern, Switzerland; 2 Department of Visceral Surgery and Medicine, Inselspital, Bern University Hospital, University of Bern, Bern, Switzerland; 3 Department for BioMedical Research, Visceral Surgery and Medicine, University of Bern, Bern, Switzerland; 4 Institute of Tissue Medicine and Pathology, University of Bern, Bern, Switzerland; 5 Waters Corporation, Wilmslow, UK

**Keywords:** Liver, Lipid metabolism, Liver zonation, Hepatic lipid distribution, Pathway analysis, *De novo* triacylglycerol biosynthesis

## Abstract

**Background & Aims:**

Lipid metabolism plays an important role in liver pathophysiology. The liver lobule asymmetrically distributes oxygen and nutrition, resulting in heterogeneous metabolic functions. Periportal and pericentral hepatocytes have different metabolic functions, which lead to generating liver zonation. We developed spatial metabolic imaging using desorption electrospray ionisation mass spectrometry to investigate lipid distribution across liver zonation with high reproducibility and accuracy.

**Methods:**

Fresh frozen livers from healthy mice with control diet were analysed using desorption electrospray ionisation mass spectrometry imaging. Imaging was performed at 50 μm × 50 μm pixel size. Regions of interest (ROIs) were manually created by co-registering with histological data to determine the spatial hepatic lipids across liver zonation. The ROIs were confirmed by double immunofluorescence. The mass list of specific ROIs was automatically created, and univariate and multivariate statistical analysis were performed to identify statistically significant lipids across liver zonation.

**Results:**

A wide range of lipid species was identified, including fatty acids, phospholipids, triacylglycerols, diacylglycerols, ceramides, and sphingolipids. We characterised hepatic lipid signatures in three different liver zones (periportal zone, midzone, and pericentral zone) and validated the reproducibility of our method for measuring a wide range of lipids. Fatty acids were predominantly detected in the periportal region, whereas phospholipids were distributed in both the periportal and pericentral zones. Interestingly, phosphatidylinositols, PI(36:2), PI(36:3), PI(36:4), PI(38:5), and PI(40:6) were located predominantly in the midzone (zone 2). Triacylglycerols and diacylglycerols were detected mainly in the pericentral region. *De novo* triacylglycerol biosynthesis appeared to be the most influenced pathway across the three zones.

**Conclusions:**

The ability to accurately assess zone-specific hepatic lipid distribution in the liver could lead to a better understanding of lipid metabolism during the progression of liver disease.

**Impact and Implications:**

Zone-specific hepatic lipid metabolism could play an important role in lipid homoeostasis during disease progression. Herein, we defined the zone-specific references of hepatic lipid species in the three liver zones using molecular imaging. The *de novo* triacylglycerol biosynthesis was highlighted as the most influenced pathway across the three zones.

## Highlights

• We characterised the hepatic lipid distribution across liver zonation using DESI-MSI.
• Fatty acids were predominantly detected in the periportal region, whereas triacylglycerols were detected mainly in the pericentral region.
• Although phospholipids were distributed in the periportal and pericentral zones, phosphatidylinositols were located predominantly in the midzone.
• *De novo* triacylglycerol biosynthesis appeared to be the most influenced pathway across the three zones.
• Assessing zone-specific hepatic lipid metabolism may lead to a better understanding of lipid homeostasis during disease progression.

## Graphical abstract

**Figure:**
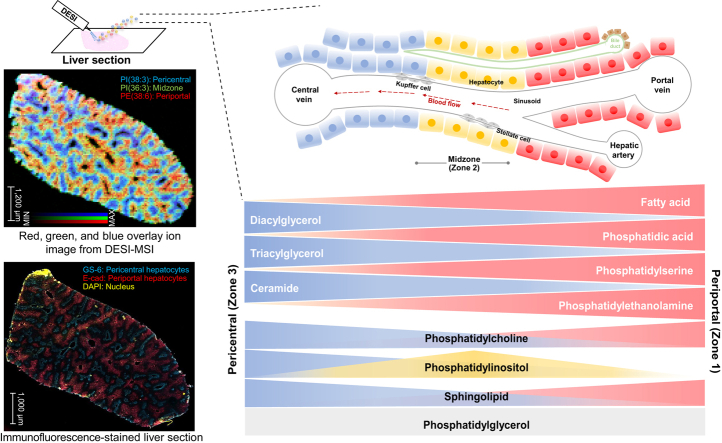

## Introduction

**Fig. 1:**
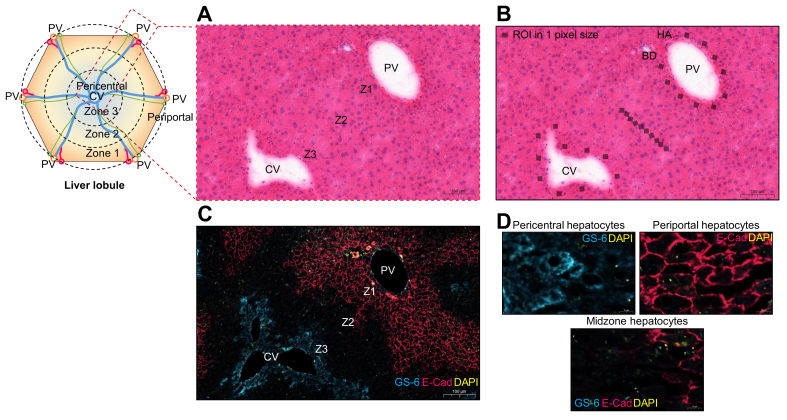
The histology guideline from H&E and double immunofluorescence staining for classification of liver zonation and ROIs drawing by co-registration with ion image. (A) H&E staining on the liver tissue. (B) The 10 pixels of each region was used to quantify the amount of lipids. (C) Double immunofluorescence staining with liver zone-specific markers. GS-6, pericentral hepatocytes (blue); E-Cad, periportal hepatocytes (red); and DAPI (yellow). (D) The hepatocytes stained in the periportal zone (Z1), midzone (Z2), and pericentral zone (Z3). BD, bile duct; CV, central vein; E-Cad, E-cadherin; GS-6, glutamine synthetase; HA, hepatic artery; PV, portal vein; ROI, region of interest; Z1, zone 1; Z2, zone 2; Z3, zone 3.

The liver architecture is built from thousands of hexagonal structures called hepatic lobules, composed of a central vein in the middle and hexagon corner of portal tracts. The hepatic lobule displays a gradient of oxygen and nutrient levels across the hepatocyte along the sinusoid from the periportal area to the pericentral site, resulting in asymmetrically distributed metabolic functions and generating a pattern known as liver zonation.^1^^,^^2^ Liver zone 1 is located in the periportal region, continued with the intermediate zone in the middle of the lobule called zone 2 and the pericentral region referred to as zone 3 (Fig. 1). Different liver zonation exhibits different metabolic and biochemical pathways, including xenobiotic, amino acid, carbohydrate, and lipid metabolism. Although the differences in metabolic zonation have been reported previously, our understanding of zone-specific lipid metabolism in the liver is limited. The heterogeneity function of fatty acid (FA) metabolism in hepatocytes isolated from different liver zones has been observed previously. The FA oxidation predominantly occurs in the periportal hepatocytes, whereas the pericentral hepatocytes showed higher rates of lipogenesis.^3^ A recent study reported the differentially expressed genes involved in FA degradation in periportal hepatocytes,^4^ whereas cholesterol and bile acid metabolism were found in pericentral hepatocytes.^4^^,^^5^ In addition, zone-specific proteomics data from isolated hepatocytes revealed a higher rate of FA uptake and FA synthesis in periportal hepatocytes.^6^ However, few studies have investigated and characterised the zone-specific hepatic lipid signature.

Several mass spectrometry (MS)-based lipidomic studies reported a wide range of lipid species in the liver using various techniques, including direct infusion (DIMS),^7^ gas chromatography,^8^ or liquid chromatography.^9^ Although these techniques are well established and allow good coverage of lipid species in the liver homogenate, they lack the resolution to differentiate the zonation of lipid metabolism in the liver. MS-based spatial metabolic imaging could provide a unique opportunity to assess zone-specific lipid metabolism in the liver. Recent studies have successfully characterised lipid signatures in the liver using MS-based imaging, including the secondary ion MS (SIMS)^10^ and matrix-assisted laser desorption/ionisation (MALDI)^11^^,^^12^ techniques. Even though both SIMS and MALDI are widely used for spatial metabolomics studies, both require sample preparation and ion generation in the high vacuum region of MS.^13^^,^^14^ The matrix choice applied for MALDI preparation is a crucial step influencing ion production; thus, matrix heterogeneity can affect the spatial integrity of the tissue surface.^15^ Furthermore, the application of SIMS imaging is limited because of the low production yield of secondary ions^16^ and the high energy of ion beam, resulting in the extensive fragmentation of molecular ions.^17^^,^^18^

A MS imaging (MSI) technique called desorption electrospray ionisation (DESI) is an ambient ionisation technique that analyses the spatial distribution and localisation of metabolites directly from tissue samples with simple sample preparation and without damaging tissue or cellular morphology,^19^^,^^20^ making it possible to perform further histology analysis. The potential of DESI-MSI for assessing lipid metabolism in different physiological conditions such as breast cancer,^19^ pancreatic cancer,^21^ brain tumour,^20^ prostate cancer,^22^ ovarian cancer,^23^ and non-small cell lung cancer^24^ has been demonstrated. DESI-MSI analysis in liver adenocarcinoma showed that unsaturated FAs containing phospholipids are predominant in the non-tumour region, whereas sphingomyelin (SM) is elevated in the tumour region.^25^ Here, we aim to develop spatial DESI-MSI to characterise hepatic lipid metabolism across the liver zonation. Understanding the role of zone-specific hepatic lipid metabolism in the normal liver is crucial to understand the metabolic changes during the disease stage.^14^ We believe this technique provides the opportunity to differentiate lipid metabolism in different liver zonation with high accuracy.

## Materials and methods

### Liver tissue collection

Five livers were collected from 16-week-old healthy male C57Bl6/J mice (Charles River, Freiburg, Germany). The 8-week-old mice were acclimatised to the housing facility for 1 week. They were housed under controlled temperature (22 ± 2 °C) and 12-h light–dark cycles. They were fed a standard diet (catalogue 5053, LabDiet, St. Louie, MO, USA). Their health, body weight, and food intake were monitored weekly.

Mice were weighed, anesthetised with pentobarbital (100 mg/kg, i.p.), and euthanised in the afternoon. The liver was collected, snap-frozen, and stored at -80 °C. All the experiments were conducted according to the regulations of the Bern Animal Welfare Committee, Canton of Bern, Switzerland (BE42/19).

### Tissue preparation

The frozen liver was sectioned at 10-μm thickness under -21 °C using the HYRAX C60 Cryostat machine (Zeiss, Jena, Germany) after mounting with a drop of distilled water. The tissue slices were thaw-mounted on glass slides (Thermo Scientific™, Waltham, MA, USA) and stored at -80 °C until DESI-MSI analysis.

The slides were dried for 15 min at room temperature before DESI-MSI analysis. The optical image was created for each slide using a CanoScan LiDE 210 scanner (Canon, Tokyo, Japan), and the slide was placed on a DESI two-dimensional moving stage holder.

### DESI-MSI analysis setup and parameters

Spatial lipid imaging data were performed using a 2D Omni Spray stage (Prosolia, Zionsville, IN, USA) coupled with Xevo™ G2-XS QTof (Waters Corporation, Wilmslow, UK). DESI-MSI data were acquired in positive and negative ionisation mode over the mass range of m/z 100 to 1,200. The imaging experiments were performed at 50-μm^2^ pixel size. The High-Performance DESI Sprayer incidence angle was set up at 75°. The distance between the sprayer and the tissue surface was 2 mm, and that between the sprayer and the inlet was 1 mm. The charged spray solvent was made of 98% methanol (Biosolve Chimie, Dieuze, France) and 2% Milli-Q water (Merck Milli-Q, Darmstadt, Germany). The solvent was sprayed at a flow rate of 2.0 μl/min. The sampling cone voltage was set up at 120 and 110 V for positive and negative ionisation, respectively. The nebulising gas (nitrogen) was set to 8.5 psi. The collection angle of the heated transfer line inlet was 10° with a distance of 0.5 mm from the tissue surface.

### Histology analysis

The classification of liver zonation was performed directly on the liver tissue used for DESI-MSI analysis by an experienced pathologist. The tissue was stained using the standard H&E staining protocol. The stained tissue sections were scanned using a Panoramic 250 Flash II slide scanner with a 20× objective (3DHISTECH Ltd., Budapest, Hungary) to generate histology scans.

### Immunofluorescence staining

Frozen liver slides were fixed for 5 min with 4% paraformaldehyde and washed with Bond™ wash solution (Leica Biosystem Newcastle Ltd, Newcastle, UK). The heating antigen retrieval was applied using EDTA/Tris and blocked with Opal™ blocking buffer (Akoya Biosciences, Marlborough, MA, USA). They were stained by glutamine synthetase (1:10,000; Sigma-Aldrich, St. Louie, MO, USA; catalogue G2781), E-cadherin (1:200; Santa Cruz Biotechnology, Inc., Dallas, TX, USA; catalogue sc-7870) antibodies, and DAPI. Then they were washed and mounted with ProLong™ Gold Antifade reagent (Thermo Fisher Scientific, Eugene, OR, USA). The immunofluorescence-stained slides were scanned using a Panoramic 250 Flash II slide scanner with a 20× objective (3DHISTECH Ltd.).

### Data and imaging analysis

MassLynx™ Software V4.2 (Waters Corporation) was used for data acquisition and spectrum preview. DESI ion images were visualised using the High Definition Imaging (HDI™) software V1.6 (Waters Corporation). The DESI-MSI data were imported into LipostarMSI V1.1.0b28 (Molecular Horizon srl, Bettona, PG, Italy) to perform data preprocessing. Briefly, preprocessing of the DESI-MSI data included peak alignment, profile smoothing, baseline correction, and peak picking.^26^ The dataset was normalised by total ion count. Tentative identification of lipids based on the accurate mass was accomplished by searching against LIPID MAPS databases (https://www.lipidmaps.org), and then lipid identification was confirmed by DESI tandem mass spectrometry (MS/MS) analysis.

Regions of interest (ROIs) were manually created by co-registration with histological data to determine spatial hepatic lipids using HDI™ software. The liver zonation was classified into zone 1 (periportal zone), zone 2 (midzone), and zone 3 (pericentral zone), as shown in Fig. 1A and C. Three different ROIs were selected in each liver zonation. Each region consisted of 10 pixels used to quantify the amount of lipids in each ROI (Fig. 1B). The mass list of specific ROIs was automatically created, and statistical analysis was performed to compare lipids between liver zonation. Lipid levels were measured in three ROIs. Thus, for each zone, 30 pixels was considered to assess lipid intensity in each zone. For the analysis, the medians of lipid intensity in each of the three ROIs were considered. For statistical analysis, a repeated-measure analysis of covariance was performed for each lipid with zonation (portal, midzone, or central) as a fixed effect and mouse identifier as a random effect. The Chi-square *p* value of the vein localisation term was reported. To account for multiple testing, Benjamini–Hochberg false discovery rate was estimated. All statistical analyses were performed using R version 4.0.2 (Hornik and R Core Team, https://CRAN.R-project.org).

### Pathway analysis

We performed pathway analysis on the list of 117 significantly different lipids across the three ROIs. We used the mouse metabolic pathway set from the PathBank database,^27^ which spans more than 12,000 pathways. The pathway scores were calculated based on the relative node betweenness centrality graph measure.^28^ Over-representation analysis was also carried out for additional insight into the pathways’ importance. We used the same pathway definitions and the hypergeometric test to calculate *p* values (statistical significance *p* ≤ 0.05). The background metabolite set for over-representation analysis was defined as the totality of the participating compounds in all pathways. All calculations were made using R version 4.0.2.

## Results

### Optimisation of liver sample preparation for lipid detection

The most common liver tissue used for histological analysis is the formalin-fixed, paraffin-embedded (FFPE) tissue. Initially, we tested DESI analysis using FFPE tissues with and without the deparaffinisation method. None of the lipids were reliably detectable in FFPE liver tissues; therefore, the fresh frozen tissue was used for our DESI-MSI analysis. The frozen liver tissue was embedded with the cryosection embedding material, optimal cutting temperature compound, and gelatine, which caused ion suppression especially in positive mode. Therefore, we decided to proceed without embedding material as a series of sections at 5 and 10 μm; the 5-μm sections were used as histology guidelines. The 10-μm sections were used for DESI analysis. We assessed the effect of tissue storage without embedding material on liver morphology over 7 months, and there was no significant difference during the storage period at -80 °C, as shown in Fig. S1. DESI-MSI data were acquired in positive and negative ionisation mode. Several DESI parameters, including spray solvent composition and spray angle, were optimised. The capillary voltage was adjusted every 0.05 kV from 0.5 to 0.75 kV in both positive and negative modes. The optimal voltage was at 0.6 kV, generating high intensity and signal stability in the liver tissue. The heated transfer line voltage was optimised at 11 V (369 °C) for positive ionisation and 13 V (494 °C) for negative ionisation. To reduce run time without compromising data quality, the stage velocity was increased from 50 to 200 μm/s, increasing the scan rate from 1 to 4 Hz under the spatial resolution of 50 μm. The chemical and physical properties of the spray solvent system affect the range of molecular detection by DESI-MSI.^29^ A mixture of methanol and water (1:1) is a commonly used solvent for lipid analysis.^30^ However, in our observation, DESI-MSI with methanol and water did not detect important lipid species relevant to liver metabolism, such as diacylglycerols (DGs), triacylglycerols (TGs), and cholesterol esters. A couple of studies reported that the modification of the methanol and water spray solvent with salts such as ammonium acetate^31^^,^^32^ and sodium carbonate^31^ or the addition of silver nitrate^33^ enhances the ability to detect TGs in biological samples using DESI-MSI analysis. We observed that modification of organic spray solvent by adding salts affects the sprayer capacity; thus, we immersed fresh frozen mouse liver slides in 25 mM ammonium acetate for 20 s before DESI-MSI analysis. The immersion in ammonium acetate improved triglycerides detection (m/z ≥840) in positive mode significantly. All TGs were detected as [M+Na]^+^ and [M+NH_4_]^+^ adducts, whereas [M+H]^+^ and [M+K]^+^ adducts were low abundant.

### Determination of hepatic lipid signature using DESI-MSI

**Fig. 2:**
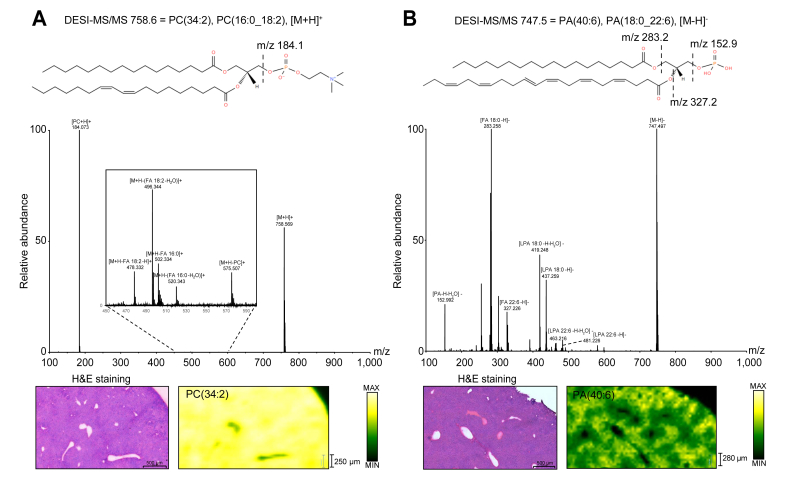
DESI-MS/MS spectrum and ion images of the healthy mouse liver obtained from (A) m/z 758.6 and (B) m/z 747.5. The lipid identification using DESI-MS/MS was confirmed as PC(16:0_18:2) and PA(18:0_22:6), respectively. The yellow colour represents the highest intensity of the lipids. DESI-MS/MS, desorption electrospray ionisation tandem mass spectrometry; FA, fatty acid; PA, phosphatidic acid; PC, phosphatidylcholine.

Our method detected 10,546 features in positive and negative ionisation modes. All mass to charge ratio (m/z, a ratio of an ion mass to its ion charge), which led to isotope pattern matching, were identified by searching against the LIPID MAPS database, and then they were confirmed by fragmentation pattern using DESI-MS/MS analysis. We successfully identified 269 lipids from the liver tissue. An example of MS/MS fragmentation using DESI-MSI is shown in Fig. 2. The m/z 758.6 and m/z 747.5 values were confirmed to be protonated adduct [M+H]^+^ of PC(16:0_18:2) and deprotonated [M-H]^-^ of PA(18:0_22:6), respectively. [M+H]^+^ and [M+K]^+^ are the most commonly reported adducts in positive mode and [M-H]^-^ adducts in negative mode.^34^ In our method, the tissue preparation generated [M+H]^+^, [M+Na]^+^, and [M+NH_4_]^+^ as the main adducts, which is similar to adducts commonly found in DIMS^7^^,^^35^ or liquid chromatography mass spectrometry (LC-MS)^9^ lipidomic analysis. The DESI-MSI method detected a broad spectrum of lipid species in the liver, including FAs, lysophospholipids (LPLs), phosphatidic acids (PAs), phosphatidylcholines (PCs), phosphatidylethanolamines (PEs), phosphatidylglycerols (PGs), phosphatidylinositols (PIs), phosphatidylserines (PSs), DGs, TGs, ceramides (Cers), and sphingolipids (SLs). The ion images in Fig. S2 show that the most abundant PC detected in the liver was PC(34:2) (m/z 758.6), which agrees with that detected by MALDI in a previous study.^11^ This was followed by PC(36:4) (m/z 782.6) and PC(38:6) (m/z 806.6). We detected FA(18:2) as the most abundant FA in the liver, which was significantly expressed in the periportal zone. At the same time, PI(38:4) (m/z 885.6) was the most abundant phospholipid, which was significantly higher in the pericentral zone.

### Validation of DESI-MSI lipidomics in liver

The reproducibility of the method was assessed using intraday and interday analysis. The results are shown in Table S1. Most detected lipids had intraday and interday deviation lower than 20%, according to the FDA guidance for bioanalytical method validation and study sample analysis. Only the low-abundant lipids showed high variation (percent coefficient of variation = 20–37%). To assess the reproducibility of the detected lipids, 10 pixels was selected for each region to assess the analytical variability. Table S2 shows the reproducibility of DESI-MSI in detecting lipids in different zones.

### Hepatic lipid distribution across liver zonation

**Fig. 3:**
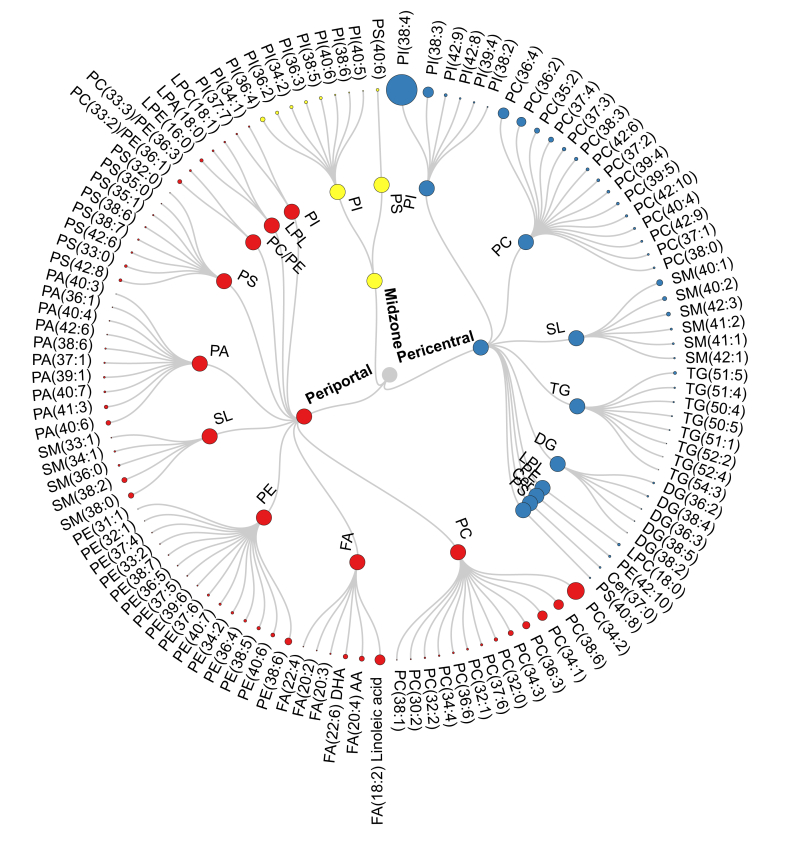
Circular dendrogram of the statistically significant lipids predominantly expressed in three liver zones: zone 1 (periportal zone; red), zone 2 (midzone; yellow), and zone 3 (pericentral zone; blue). AA, arachidonic acid; Cer, ceramide; DG, diacylglycerol; DHA, docosahexaenoic acid; FA, fatty acid; LPA, lysophosphatidic acid; LPC, lysophosphatidylcholines; LPE, lysophosphatidylethanolamine; LPL, lysophospholipid, PA, phosphatidic acid; PC, phosphatidylcholine; PE, phosphatidylethanolamine; PI, phosphatidylinositol; PS, phosphatidylserine; SL, sphingolipid; TG, triacylglycerol.

**Fig. 4:**
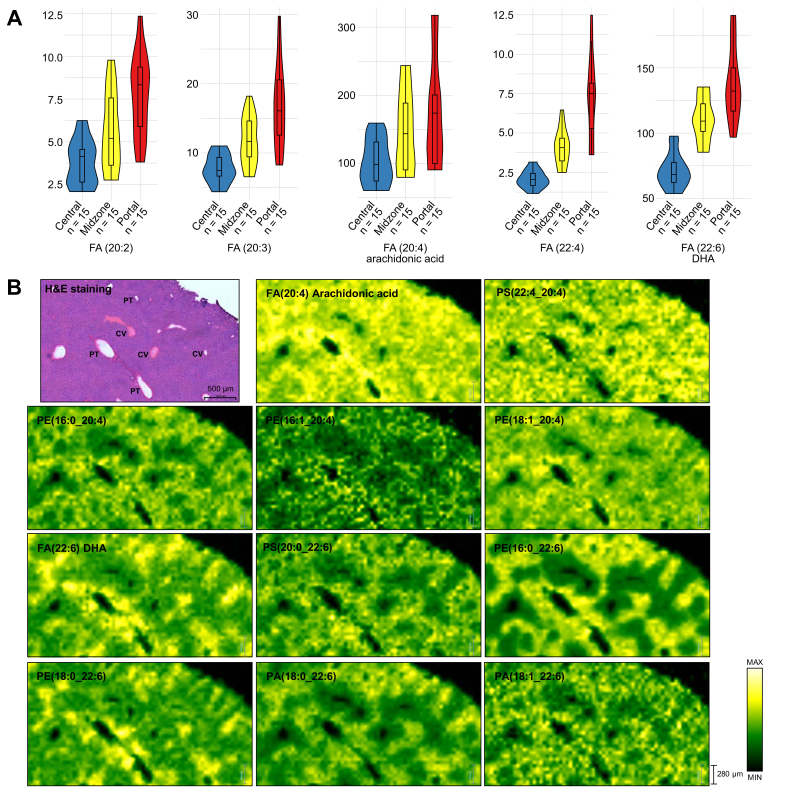
FAs and phospholipids containing n-6 PUFA arachidonic acid and n-3 PUFA DHA distribution were observed in the healthy liver. Statistical analysis was performed to compare FAs distributed across three liver zones (periportal zone [red], midzone [yellow], and pericentral zone [blue]), statistical significant *p* ≤ 0.05. (A) The violin boxplots show that FA(20:2), FA(20:3), FA(20:4), FA(22:4), and FA(22:6) were detected predominantly in the portal tracts. (B) DESI-MSI ion images of arachidonic acid FA(20:4), DHA FA(22:6), phospholipids containing arachidonic acid, and phospholipids containing DHA distribute across liver zonation and highly express in the periportal zone. The yellow colour represents the highest intensity of the lipids. CV, central vein; DESI-MSI, desorption electrospray ionisation mass spectrometry imaging; DHA, ocosahexaenoic acid; FA, fatty acid; PA, phosphatidic acid; PE, phosphatidylethanolamine; PS, phosphatidylserine; PT, portal tracts; PUFA, polyunsaturated fatty acid.

**Fig. 5:**
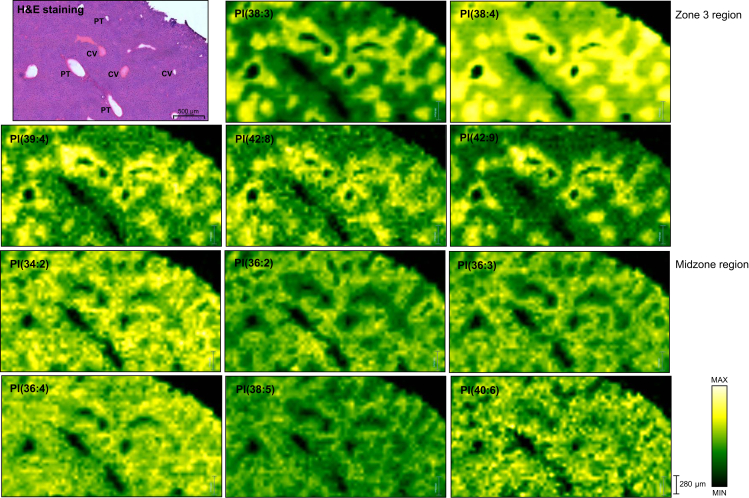
Spatial distribution of PIs obtained from healthy mice show the localisation of PI(38:3), PI(38:4), PI(39:4), PI(42:8), and PI(42:9) in the pericentral zone of liver lobule, whereas PI(34:2), PI(36:2), PI(36:3), PI(36:4), PI(38:5), and PI(40:6) were mainly located in the midzone. The yellow colour represents the highest intensity of the lipids. CV, central vein; PI, phosphatidylinositol; PT, portal tracts.

**Fig. 6:**
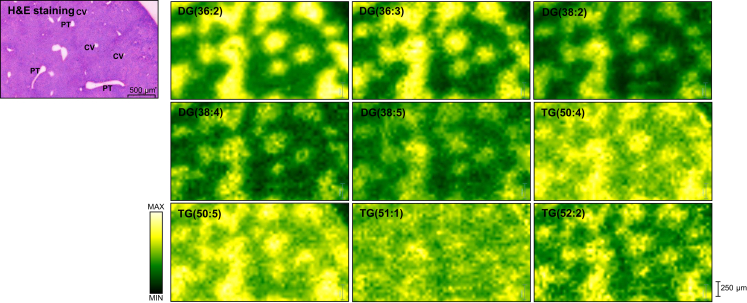
DESI-MSI ion images of DG(36:2), DG(36:3), DG(38:2), DG(38:4), DG(38:5), TG(50:4), TG(50:5), TG(51:1), and TG(52:2) show the similar distribution patterns of diacylglycerols and triacylglycerols in the healthy mice liver. The distribution of these lipids was predominantly detected in the pericentral zone. The yellow colour represents the highest intensity of the lipids. CV, central vein; DG, diacylglycerol; PT, portal tracts; TG, triacylglycerol.

Spatial metabolic imaging sheds light on the distribution of hepatic lipids across the liver zonation. Statistically significant expressed lipids across liver zonation are illustrated in Fig. 3 and Table S3. Debois *et al.*^10^ detected FA(16:1) outside of steatotic vesicles, FA(18:1) in the intermediate localisation, and FA(18:2) in lipid droplets. However, the localisation of these FAs and the others in the liver lobule has not been previously described. In our study, polyunsaturated FAs (PUFAs) including FA(18:2), FA(20:2), FA(20:3), arachidonic acid (FA(20:4)), FA(22:4), and docosahexaenoic acid (DHA) (FA(22:6)) were significantly higher in the periportal region (Fig. 3, Fig. 4A). Interestingly, the phospholipids containing arachidonic acid PS(22:4_20:4), PE(16:0_20:4), PE(16:1_20:4), and PE(18:1_20:4) were also significantly higher in the periportal region than in other zones (Fig. 4B). This pattern was also observed among the phospholipids containing DHA, such as PS(20:0_22:6), PE(16:0_22:6), PE(18:0_22:6), PA(18:0_22:6), and PA(18:1_22:6), which were expressed predominantly in the periportal region as shown in Fig. 4B. PCs were the predominant lipid species in healthy mouse liver. Interestingly, we observed that the distribution of PCs in different zone was highly related to their FA composition. As shown in Fig. S3A, PC(32:1), PC(34:1), PC(34:2), PC(34:3), PC(34:4), PC(36:3), PC(36:6), PC(37:6) and PC(38:6) were strongly located in the periportal region, whereas PC(35:2), PC(36:2), PC(36:4), PC(37:1), PC(37:2), PC(37:3), PC(38:0), PC(39:5), and PC(40:4) were significantly more expressed in the pericentral region (Fig. S3B). Heterogeneous distribution was also observed in PIs (Fig. 5); most PIs, such as PI(38:3), PI(38:4), PI(39:4), PI(42:8), and PI(42:9), were detected mainly in the pericentral region, whereas PI(34:2), PI(36:2), PI(36:3), PI(36:4), PI(38:5), PI(38:6), PI(40:5), and PI(40:6) were mainly distributed in the midzone (zone 2). As shown in Fig. S4**,** LPLs such as lysophosphatidic acid (LPA) (18:0), lysophosphatidylethanolamine (16:0), and lysophosphatidylcholines (LPC) (18:1) were concentrated in periportal areas, whereas LPC(18:0) was localised in pericentral areas. PGs did not show a significant difference in distribution across the zonation. DGs and TGs exhibited similar patterns; as shown in Figure 6, both lipid classes were predominantly present in the pericentral region. Furthermore, we detected Cer and several SLs across liver zonation (Fig. S5). Cer(37:0) was significantly higher in the pericentral region. Although most of the SLs were mainly expressed in the pericentral region, SM(36:0), SM(38:0), and SM(38:2) were significantly higher in the periportal region.

**Fig. 7:**
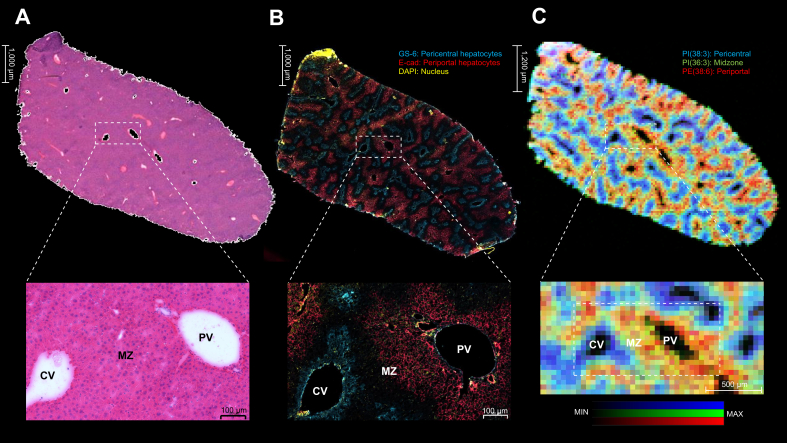
Comparison of the liver zonation pattern using liver histology, immunofluorescence imaging, and molecular imaging with DESI. (A) H&E staining of liver tissue after DESI-MSI analysis. (B) Double immunofluorescence staining shows the proto-central axis along with the liver zonation. GS-6, pericentral hepatocytes (blue); E-Cad, periportal hepatocytes (red); and DAPI (yellow). (C) Red, green, and blue overlay ion image from DESI-MSI: PI(38:3) located in the pericentral area (blue); PI(36:3) mainly expressed in the midzone (green); and PE(38:6) predominantly presented in the periportal region (red). CV, central vein; DESI, desorption electrospray ionisation; DESI-MSI, desorption electrospray ionisation mass spectrometry imaging; E-Cad, E-cadherin; GS-6, glutamine synthetase; MZ, midzone; PE, phosphatidylethanolamine; PI, phosphatidylinositol; PV, portal vein.

We performed double immunofluorescence staining with liver-specific markers to validate the zonation pattern of the liver. We used glutamine synthetase (GS-6), which is selectively expressed in pericentral hepatocytes, and E-cadherin (E-Cad) for periportal hepatocytes as well as DAPI to stain the hepatocytes nucleus.^36^ Fig. 7 shows the determination of liver zonation using (a) H&E staining, (b) double immunofluorescence staining, and (c) zone-specific lipid distribution using DESI-MSI.

### Identification of key metabolic pathways in hepatic zonation

**Fig. 8:**
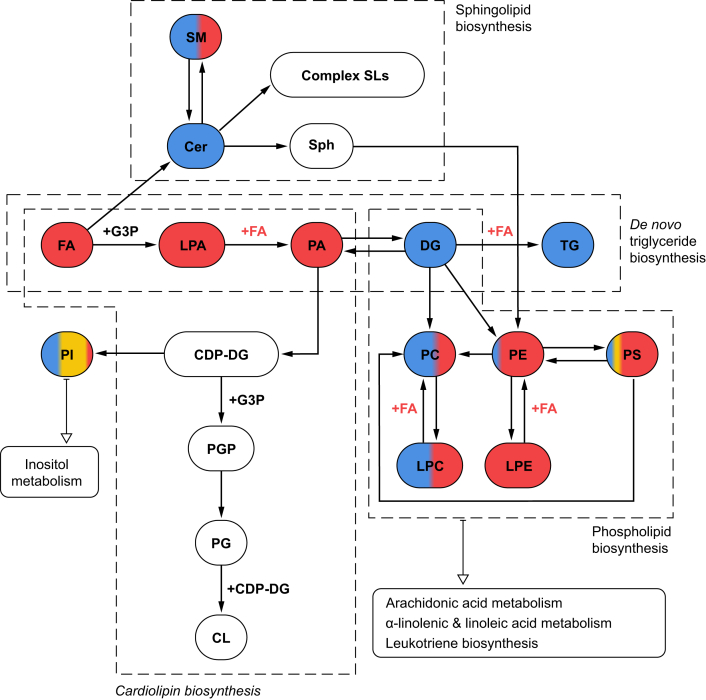
Top-ranked pathways across the three regions of interest. Colours indicate the regions in which each lipid family was predominantly detected. Multiple colours indicate that lipid classes were expressed in multiple zones. Red, periportal zone; yellow, midzone; blue, pericentral zone; white, not detected. CDP-DG, cytidinediphosphate-diacylglycerol; Cer, ceramide; CL, cardiolipin; DG, diacylglycerol; FA, fatty acid; G3P, glycerol-3-phosphate; LPA, lysophosphatidic acid); LPC, lysophosphatidylcholines; LPE, lysophosphatidylethanolamine; PA, phosphatidic acid; PC, phosphatidylcholine; PE, phosphatidylethanolamine; PG, phosphatidylglycerol; PGP, phosphatidylglycerophosphate; PI, phosphatidylinositol; PS, phosphatidylserine; SL, sphingolipid; SM, sphingomyelin; Sph, sphingosine; TG, triacylglycerol.

We performed pathway analysis to investigate the alteration in lipid metabolism across liver zonation. The top ranked pathways with their respective dominant ROI expression profiles are displayed in Fig. 8. The analysis revealed that the *de novo* TG biosynthesis is the most influenced pathway across the three regions, followed by phospholipid, cardiolipin, and SL biosyntheses. Other implicated pathways include the metabolism of inositol and phosphatidylinositol, arachidonic, alpha-linolenic, and linoleic acids, as well as leucotriene biosynthesis.

## Discussion

Several studies have reported the zonation of different biochemical pathways in the liver.[37], [38], [39] However, our understanding of zone-specific lipid metabolism is limited. MSI with minimum sample preparation, such as DESI, would facilitate investigating zone-specific lipid metabolism without impacting the integrity of liver tissue. Moreover, label-free and matrix-free molecular imaging of tissue histology is beneficial for precise assessment of lipid metabolism across liver zonation, especially in clinical settings, as it is simple and reproducible compared with other imaging techniques. Thus, we developed and validated a robust and reproducible method of spatial metabolic imaging using DESI-MS to assess hepatic lipid zonation. Our approach allows characterising a wide range of lipid species across the liver zonation with high reproducibility and accuracy. Immersion of prepared tissue in 25 mM ammonium acetate before DESI-MSI analysis improved the detection of TGs owing to the formation of ammonium adduct, which agreed with the previous report.^31^

We identified 269 lipids across the liver zonation that could be monitored reproducibly. Hepatic lipid alteration has been shown to play a crucial role in the pathogenesis of liver diseases. Several studies reported lipid levels in liver homogenates and circulation, such as serum and plasma, using various MS techniques.[7], [8], [9]^,^^35^ However, these analyses do not capture the zonation of lipid metabolism in the liver. The liver zonation may have a vital role in liver diseases; thus, assessing zone-specific hepatic lipid metabolism will improve our understanding of the pathogenesis of the liver. A couple of MS-based imaging studies using SIMS and MALDI has described the zone-specific lipids in the liver. Debois *et al.*^10^ exhibited the zonation of lipids, including FAs and DGs, in the healthy liver using SIMS. However, arachidonic acid (FA(20:4)), phospholipids, and TGs, could not be detected by SIMS. PC and PE zonation has been reported in the normal obese liver by Wattacheril *et al.*,^11^ and TG distribution has been observed by Alamri *et al.*^12^ using MALDI. However, the spatial distribution of other lipid species, such as LPLs, PAs, PSs, PIs, Cers, and SLs across liver zonation has not been investigated previously. Therefore, we investigated the localisation of 269 identified lipids, which could be measured reliably and accurately with DESI-MSI. A total of 117 lipids (Fig. 3) were significantly different across the three zones. We observed that FAs, LPLs, PAs, PCs, PEs, PSs, PIs, DGs, TGs, Cers, and SLs expressed heterogeneously across liver zonation, whereas PGs were distributed homogeneously. It is worth mentioning that despite our efforts to detect cholesterol and cholesterol esters, we were not able to detect them reliably using DESI-MSI. This is, at least in part, attributable to the low affinity and low acidity of cholesterol impact on the ionisation by DESI.^40^ This agrees with recent studies, which have reported similar challenges.^40^^,^^41^ Further investigations are required to optimise this technique for cholesterol and cholesterol ester detection.

We observed a gradient increase in FAs from the periportal to pericentral regions. This is expected as the blood flows from the portal vein and diffuses along the sinusoid to the central vein,^39^ resulting in a different FA uptake from the periportal area to the pericentral area. This agrees with previous findings indicating higher FA uptake rate as well as higher FA synthesis in periportal hepatocytes.^6^ Even though, CD36, the protein involved in FA uptake, has been predominantly detected in pericentral hepatocytes.^42^ Guzman and Castro^3^ reported a higher capacity of FA esterification into cellular lipids and VLDL in pericentral hepatocytes, which supports the low intensity of FAs detected in the pericentral area. Schleicher *et al.*^43^ used a mathematical multicompartment model of hepatic FA metabolism coupled with blood flow simulations and demonstrated that the lower oxygen supply in the pericentral zone enhanced the pericentral TG accumulation, whereas the higher rate of FA oxidation in the periportal zone was dominated by a higher oxygen supply. In support of this, another study by Bulutoglu *et al.*^44^ indicated that the lowest oxygen supply, as in the pericentral region, drove the highest TG accumulation at the lowest free FA supplementation. In agreement with the previous observation,^12^ we demonstrated that TGs were predominantly localised in the pericentral region. This may be explained by the fact that the pericentral area has a lower oxygen supply and generates a lower rate of FA oxidation.^3^ Once the FA oxidation is saturated, to avoid cytotoxicity, the excess FAs are esterified to TGs and stored as lipid droplets or secreted in the form of VLDL. Glycerophospholipids are a major lipid component of cellular membranes.^45^ Among the total cellular phospholipids, PIs are expressed around 10–20% of total phospholipids.^46^ The reliable assessment of PIs is challenging owing to their low abundance in the biological system, as well as analytical challenges such as the high polarity of the phosphate group and the acidic nature of phosphates. However, our DESI-MSI method successfully detected the PIs in deprotonated form. Most notably, we discovered that PIs, which are involved in cellular signalling, cell apoptosis,^47^ and cell proliferation,^48^ were predominantly accumulated in the midzone (zone 2). To the best of our knowledge, this has never been reported previously. Wei *et al.*^49^ reported that zone 2 might be a vital zone for liver repopulation. During homoeostasis and regeneration, zone 2 is an essential source of new hepatocytes, driven by the insulin-like growth factor binding protein 2–mechanistic target of rapamycin–cyclin D1 (IGFBP2-mTOR-CCND1) axis that might regulate phosphatidylinositol 3-kinase–mTOR signalling.

In addition, we demonstrated that the zonation pattern of specific lipids across the liver lobule corresponds to microscopic histology by performing double immunofluorescence staining (Fig. 7).

Finally, we performed pathway analysis to explore the metabolic pathways associated with liver zonation (Fig. 8). TG biosynthesis via glycerol-3-phosphate (G3P), appeared to be the most zone-influenced pathway. Halpern *et al.*^5^ and Ben-Moshe *et al.*^42^ showed that around 50% of liver genes exhibited zone-specific expression in the liver lobule; however, they only reported acylglycerol-3-phosphate acyltransferases (Agpat2 and Agpat3) protein expressed significantly in the pericentral zone.^42^ In addition, phospholipid and cardiolipin biosynthesis were very highly ranked in our pathway analysis. This is potentially as a result of the involvement of FAs, PA, and LPA as the major precursors in cardiolipin synthesis and remodelling.^50^

The main goal of the current study was to develop a spatial molecular imaging approach to elucidate lipid metabolism across liver zonation and explore the hepatic lipid signatures in the healthy liver. The simple sample tissue preparation for DESI-MSI analysis (label-free, matrix-free, and without damaging tissue or cellular morphology) allows for performing co-registration with ion images and histological data directly on the tissue used for the DESI-MSI analysis without further steps. This is beneficial for clinical studies where the availability of liver biopsies is limited. The ability to perform spatial liver imaging with high accuracy and precision will lead to a better understanding of zone-specific hepatic lipid metabolism, especially in chronic liver diseases such as non-alcoholic fatty liver disease progression. Further research could explore the alteration of lipid distribution across the liver zonation in liver diseases such as non-alcoholic fatty liver disease or investigate the zonation adaptation during the circadian rhythm.

## Financial support

JFD and MoM received funding from Waters Corporation. MoM received funding from the Swiss National Science Foundation (SNSF), Switzerland (grant nos. 190686 and 213352).

## Authors’ contributions

Developed the concept and designed the study: MoM, JFD. Developed and performed experiments: PS, MaM. Supported the method development: MoM, EC, AP, UR. Analysed and interpreted the data: PS, MoM. Performed pathway analysis: ST. Drafted the original manuscript: PS and MoM. Critically revised, contributed, and approved the final version of the manuscript: all authors.

## Data availability statement

The authors confirm that the data supporting the findings of this study are available within the article and supplementary material. Due to the confidentiality of data, other than those is only available on request to the corresponding author (MoM).

## Conflicts of interest

EC is an employee of Waters Corporation. The remaining authors have no conflicts of interest to declare.

Please refer to the accompanying ICMJE disclosure forms for further details.
