# Supplementary material for: Characterisation of hepatic lipid signature distributed across the liver zonation using mass spectrometry imaging
Source: JHEP Rep. 2023 Mar 9;5(6):100725. doi: 10.1016/j.jhepr.2023.100725 (PMC10240278; doi:10.1016/j.jhepr.2023.100725)
Supplement: Multimedia component 1 [file mmc1.pdf]

Characterization of hepatic lipid signature distributed across the liver zonation using mass spectrometry imaging

Patcharamon Seubnooch, Matteo Montani, Sofia Tsouka, Emmanuelle Claude, Umara Rafiqi, Aurel Perren, Jean-Francois Dufour, Mojgan Masoodi

Supplemental Tables

Table S1. Intraday and interday analysis in mice liver using DESI-MSI

Table S2. The precision and reproducibility of all lipids detected by DESI-MSI analysis in three liver zonation

Table S3. The list of lipids significantly altered across liver zonation

Supplemental Fig.s

..

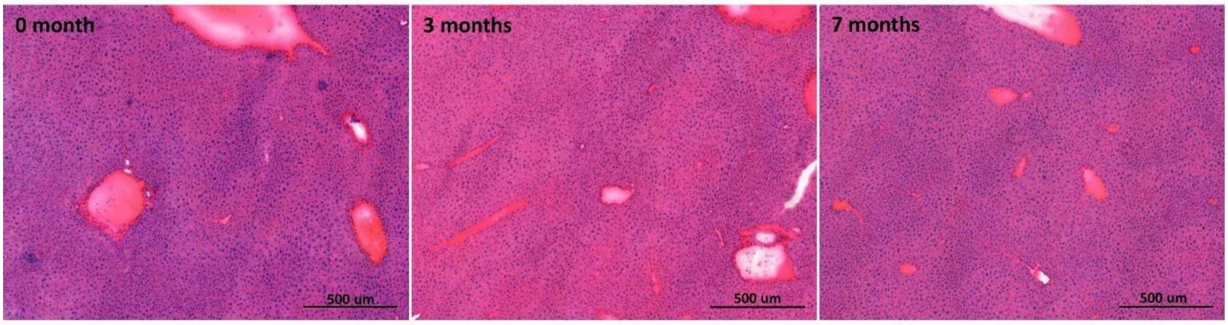

Fig. S1 The H&E staining of liver tissue after storage at -80 °C. The staining showed the comparison of the tissue morphology after sectioning without embedding material and stored at -80 °C for 0, 3, and 7 months.

..

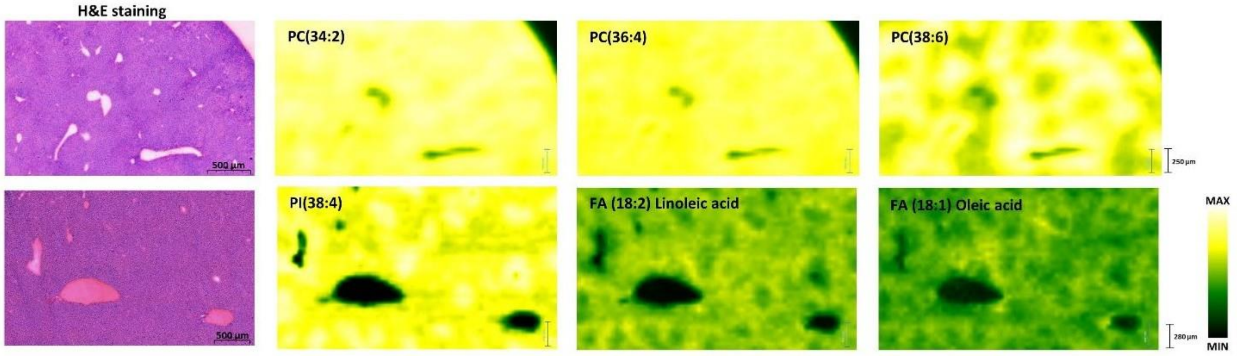

Fig. S2 The most abundant lipids detected by DESI-MSI analysis. The ion images showed that PC(34:2), PC(36:4), and PC(38:6) were exposed with the highest intensity in positive ionization mode. FA(18:2) or linoleic acid and FA(18:1) or oleic acid and PI(38:4) are the major lipids detected in negative ionization mode. The yellow color represents the highest intensity of the lipids.

..

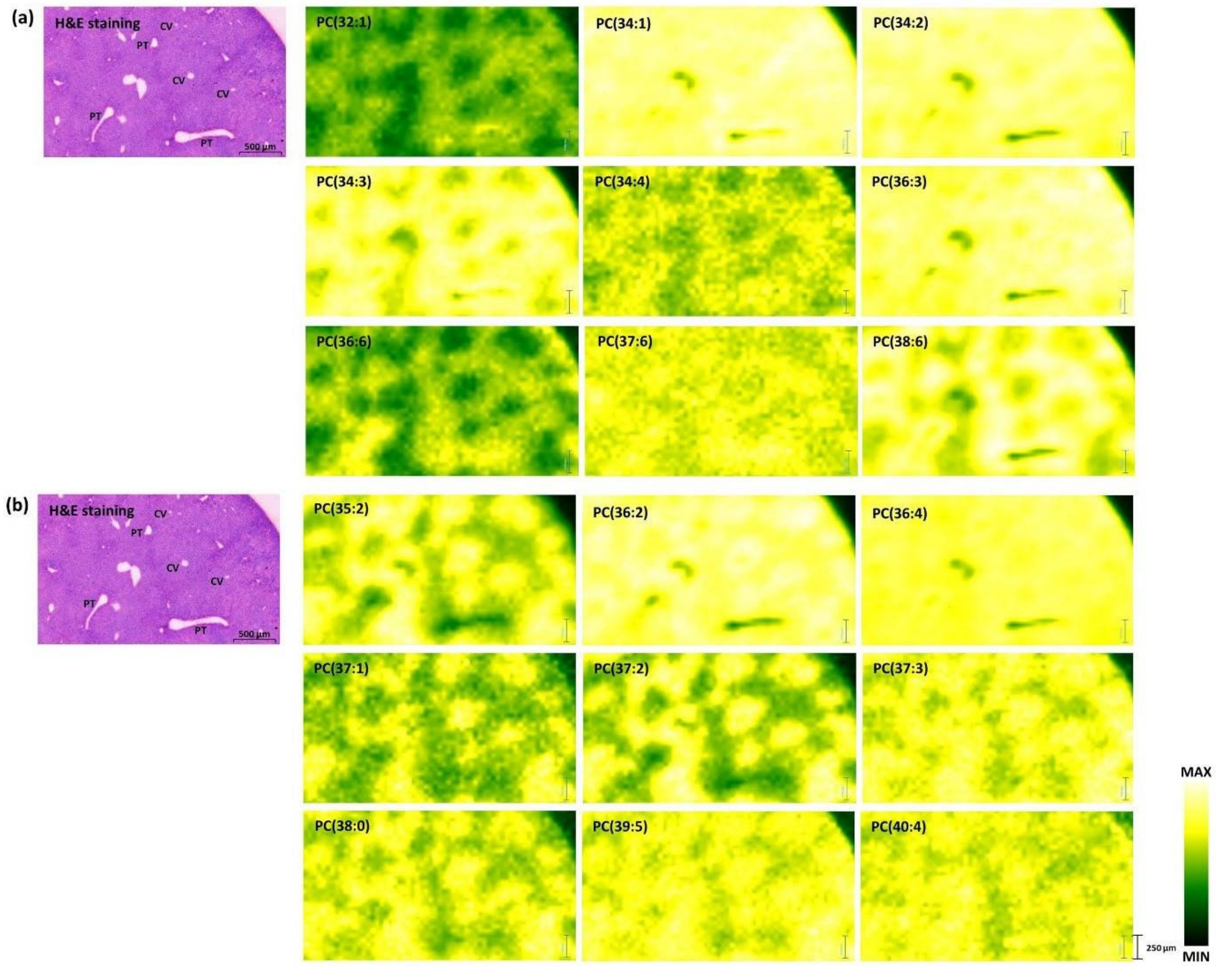

Fig. S3 The heterogeneous distribution of phosphatidylcholines in liver tissues, PC(32:1), PC(34:1), PC(34:2), PC(34:3), PC(34:4), PC(36:3), PC(36:6), PC(37:6) and PC(38:6) were strongly located in periportal region (a) whereas PC(35:2), PC(36:2), PC(36:4), PC(37:1), PC(37:2), PC(37:3), PC(38:0), PC(39:5), and PC(40:4) were predominantly expressed in pericentral region (b). The yellow color represents the highest intensity of the lipids. CV, central vein; PT, portal tracts.

..

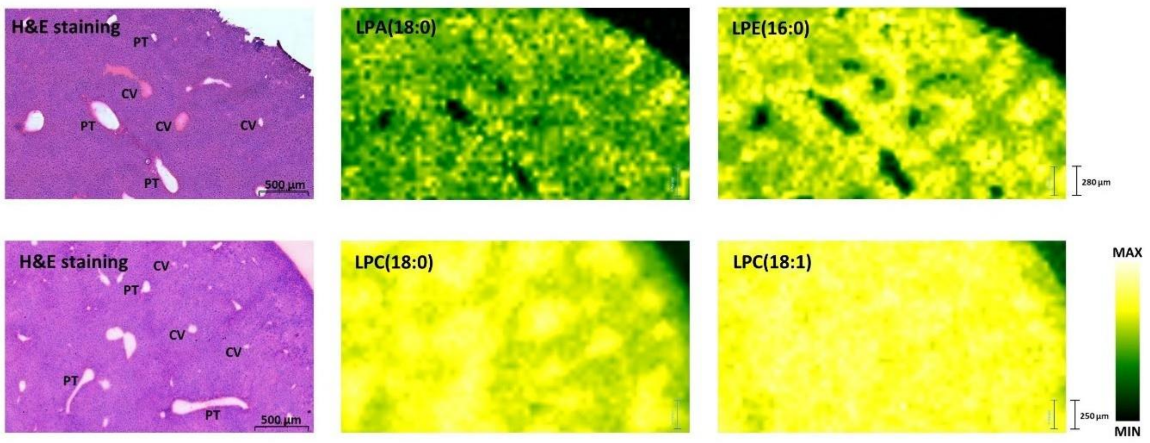

Fig. S4 Significantly distribution of lysophospholipids across liver zonation. LPA(18:0), LPE(16:0), and LPC(18:1) were mainly located in periportal areas, whereas LPC(18:0) was localized in pericentral areas. The yellow color represents the highest intensity of the lipids. CV, central vein; PT, portal tracts.

..

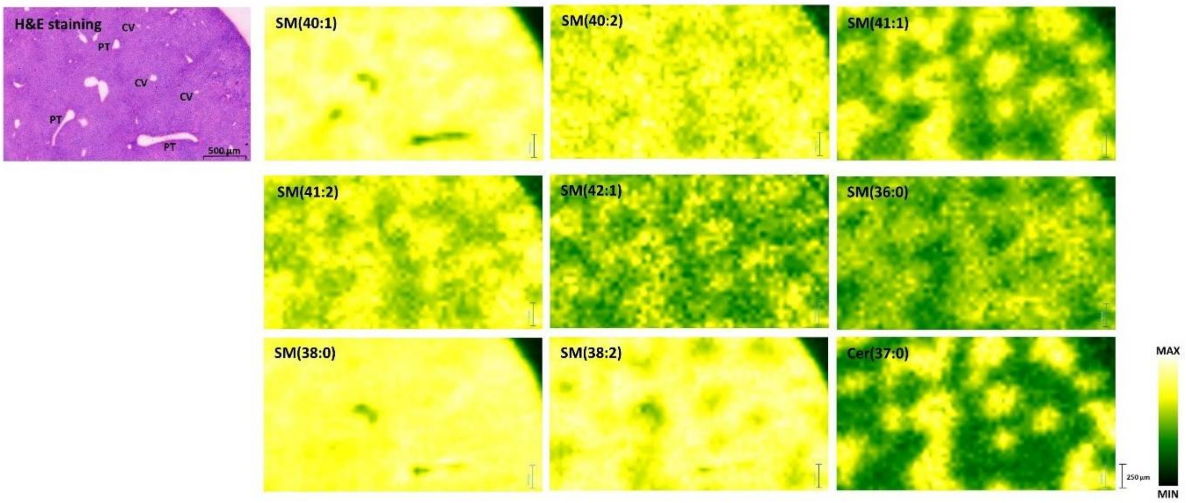

Fig. S5 The localization of ceramides and sphingolipids in healthy mice liver. Cer(37:0) was significantly higher in the pericentral region. SM(40:1), SM(40:2), SM(41:1), SM(41:2), and SM(42:1) were mainly expressed in the pericentral region, whereas SM(36:0), SM(38:0), and SM(38:2) were significantly higher in the periportal region. The yellow color represents the highest intensity of the lipids. CV, central vein; PT, portal tracts.

..

Supplemental Methods

Histology defining on liver tissue

The classification of liver zonation and defined ROIs using H&E staining based on the microscopic architecture of liver tissue to describe the microanatomy of the liver[S1]. The midzone was defined according to the concept of the liver lobular and liver acinus. As shown in Supplement method Fig. 1, we measured the distance between a portal tract and a central vein and divided them into three parts. Zone 1 is the area close to the portal vein consisting of 6-8 hepatocytes, was defined as a periportal zone, the location close to the central vein was indicated as a pericentral area, and the midzone was the intermediate area between the periportal and pericentral zones consisting of 6-10 hepatocytes[S2].

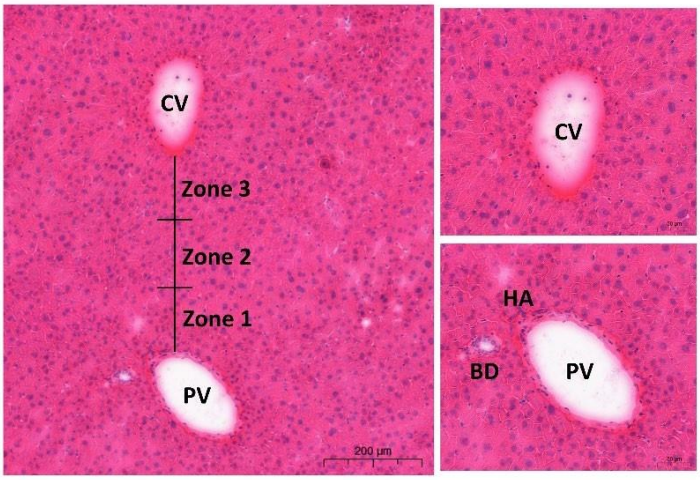

Supplement method Fig. 1: The H&E staining showed the division of liver tissue into three zones. The classification of liver zonation; Zone 1, periportal zone; Zone 2, midzone; Zone 3, pericentral zone. CV, central vein; PV, portal vein; BD, bile duct; HA, hepatic artery.

..

Supplemental References

S1. Krishna, M., Microscopic anatomy of the liver. *Clin Liver Dis (Hoboken)* **2013**, 2 (Suppl 1), S4-S7.

S2. Ma, R.; Martinez-Ramirez, A. S.; Borders, T. L.; Gao, F.; Sosa-Pineda, B., Metabolic and non-metabolic liver zonation is established non-synchronously and requires sinusoidal Wnts. *Elife* **2020,** *9*.

..
