# Supplementary material for: Characterisation of hepatic lipid signature distributed across the liver zonation using mass spectrometry imaging
Source: JHEP Rep. 2023 Mar 9;5(6):100725. doi: 10.1016/j.jhepr.2023.100725 (PMC10240278; doi:10.1016/j.jhepr.2023.100725)
Supplement: Multimedia component 5 [file mmc5.docx]

**Journal of Hepatology**

**CTAT methods**

Tables for a “Complete, Transparent, Accurate and Timely account” (CTAT) are now mandatory for all revised submissions. The aim is to enhance the reproducibility of methods.

- Only include the parts relevant to your study
- Refer to the CTAT in the main text as ‘Supplementary CTAT Table’
- Do not add subheadings
- Add as many rows as needed to include all information
- Only include one item per row

**If the CTAT form is not relevant to your study, please outline the reasons why:**

|  |
| --- |

- 1. **Antibodies**

| **Name** | **Citation** | **Supplier** | **Cat no.** | **Clone no.** |
| --- | --- | --- | --- | --- |
| **Glutamine synthetase (GS-6)** | **https://doi.org/10.7554/eLife.46206** | **Sigma-Aldrich** | **G2781** |  |
| **E-cadherin (E-Cad)** | **https://doi.org/10.7554/eLife.46206** | **Santa Cruz Biotechnology, Inc.,** | **sc-7870** |  |

- 1. **Cell lines**

| **Name** | **Citation** | **Supplier** | **Cat no.** | **Passage no.** | **Authentication test method** |
| --- | --- | --- | --- | --- | --- |
|  |  |  |  |  |  |

- 1. **Organisms**

| **Name** | **Citation** | **Supplier** | **Strain** | **Sex** | **Age** | **Overall n number** |
| --- | --- | --- | --- | --- | --- | --- |
| **Mouse** |  | **Charles River, Freiburg, Germany** | **C57Bl6/J** | **male** | **8 weeks** | **5** |

- 1. **Sequence based reagents**

| **Name** | **Sequence** | **Supplier** |
| --- | --- | --- |
|  |  |  |

- 1. **Biological samples**

| **Description** | **Source** | **Identifier** |
| --- | --- | --- |
|  |  |  |

- 1. **Deposited data**

| **Name of repository** | **Identifier** | **Link** |
| --- | --- | --- |
|  |  |  |

- 1. **Software**

| **Software name** | **Manufacturer** | **Version** |
| --- | --- | --- |
| **MassLynx™ Software** | **Waters Corporation, UK** | **V4.2** |
| **High Definition Imaging (HDI) software** | **Waters Corporation, UK** | **V1.6** |
| **LipostarMSI** | **Molecular Horizon srl, Italy** | **V1.1.0b28** |

- 1. **Other (e.g. drugs, proteins, vectors etc.)**

|  |  |  |
| --- | --- | --- |
|  |  |  |

- 1. **Please provide the details of the corresponding methods author for the manuscript:**

| **Mojgan Masoodi**  **Address: Universitätsinstitut für Klinische Chemie (UKC), Inselspital, Freiburgstrasse / INO F, 3010 Bern, Switzerland**  **Telephone: +41 31 632 21 11**  **Email: mojgan.masoodi@insel.ch** |
| --- |

**2.0 Please confirm for randomised controlled trials all versions of the clinical protocol are included in the submission. These will be published online as supplementary information.**

|  |
| --- |
